# On the Use of EEG or MEG Brain Imaging Tools in Neuromarketing Research

**DOI:** 10.1155/2011/643489

**Published:** 2011-09-27

**Authors:** Giovanni Vecchiato, Laura Astolfi, Fabrizio De Vico Fallani, Jlenia Toppi, Fabio Aloise, Francesco Bez, Daming Wei, Wanzeng Kong, Jounging Dai, Febo Cincotti, Donatella Mattia, Fabio Babiloni

**Affiliations:** ^1^Department of Physiology and Pharmacology, Sapienza University of Rome, 00185 Rome, Italy; ^2^Fondazione Santa Lucia, IRCCS, 00179 Rome, Italy; ^3^Department of Computer Science and Systems, Sapienza University of Rome, 00185 Rome, Italy; ^4^College of Computer Science, Hangzhou Dianzi University, Hangzhou 310018, China

## Abstract

Here we present an overview of some published papers of interest for the marketing research employing electroencephalogram (EEG) and magnetoencephalogram (MEG) methods. The interest for these methodologies relies in their high-temporal resolution as opposed to the investigation of such problem with the functional Magnetic Resonance Imaging (fMRI) methodology, also largely used in the marketing research. In addition, EEG and MEG technologies have greatly improved their spatial resolution in the last decades with the introduction of advanced signal processing methodologies. By presenting data gathered through MEG and high resolution EEG we will show which kind of information it is possible to gather with these methodologies while the persons are watching marketing relevant stimuli. Such information will be related to the memorization and pleasantness related to such stimuli. We noted that temporal and frequency patterns of brain signals are able to provide possible descriptors conveying information about the cognitive and emotional processes in subjects observing commercial advertisements. These information could be unobtainable through common tools used in standard marketing research. We also show an example of how an EEG methodology could be used to analyze cultural differences between fruition of video commercials of carbonated beverages in Western and Eastern countries.

## 1. Introduction

In scientific literature, the most accepted definition of neuromarketing is that it is a field of study concerning the application of neuroscientific methods to analyze and understand human behaviour related to markets and marketing exchanges [1]. Nowadays, neuroscientific methodology includes powerful brain imaging tools, based on the gathering of hemodynamic or electromagnetic signals related to the human brain activity during the performance of a relevant task for marketing objectives. The reason why marketing researchers are interested to the use of brain imaging tools, instead to simply ask to the persons their preferences in front of marketing stimuli, arises from the assumption that people cannot (or do not want) fully explain their preference when explicitly asked. 

Researchers in the field hypothesize that neuroimaging tools can access information within the consumer's brain during the generation of a preference or the observation of a commercial advertising. If this information could be useful to further promote the product is still a matter of debate in marketing literature. From the marketing researchers point of view, there is the hope that this body of brain imaging techniques will provide an efficient tradeoff between costs and benefits of the research.

The most popular brain imaging method adopted in the neuromarketing field is the functional Magnetic Resonance Image (fMRI), a technique that returns a sequence of images of the cerebral activity by means of the measure of the cerebral blood flow. Although such as images are “static”, that is, they are related to around ten seconds activity, they have a high-spatial resolution that no other neuroimaging method can offer. Nowadays, fMRI scanners are currently employed in the neuromarketing field and in the literature there exist some scientific studies showing the activation of particular cerebral areas during the tasting of a couple of popular drinks such as Coca-Cola and Pepsi [2]. 

It is very well known that the hemodynamic measurements of the brain activity allow a level of localization of the activated brain structures on the order of few cubic mm, being capable to detect activations also in deep brain structures such as amygdale and nucleus accumbens. However, the lack of time resolution, due to the delay of the cerebral blood flow's increment after the exposition to the stimuli, make the fMRI unsuitable to follow the brain dynamics on the base of its subseconds activity. 

However, there are other brain imaging techniques that allow to follow on a millisecond base the brain activity during the exposition to relevant marketing stimuli. Such techniques are the electroencephalography (EEG) and the magnetoencephalography (MEG). One problem of such brain imaging techniques is that the recorded electrical or magnetic cerebral signals are mainly due to the activity generated on the cortical structures of the brain. In fact, the electromagnetic activity elicited by deep structures (usually advocated for the generation of emotional processing in humans) is almost impossible to gather from usual superficial EEG electrodes or MEG sensors [3, 4]. To overcome this problem, high-resolution EEG technology has been developed to enhance the poor spatial information content of the EEG activity in order to detect the brain activity with a spatial resolution of a squared centimetre and the unsurpassed time resolution of milliseconds [3, 5–8]. 

On the other hand, by using the MEG signals it is also possible to estimate the activity at the level of brain voxels by using the magnetic field tomography (MFT) that it is the name given to the resulting method which perform the estimation of brain activity [9]. MFT solutions can scrutinize brain function at multiple spatiotemporal scales. In the spatial domain, the range covers details of a few millimetres allowing to map almost the entire brain. In the time domain, events can be analyzed at time scales from a fraction of a millisecond to minutes and hours [10]. 

It is worth noting that EEG and MEG techniques, while exhibiting a remarkable time resolution and a passable spatial resolution, have drastically different costs for the marketing research. In fact, MEG technology uses liquid helium and needs special shielded structures in order to record the tiny brain magnetic signals produced of the order of femtotesla. On the contrary, EEG devices are relatively inexpensive, robust, and even wearable by the subject, making such EEG technology of interest for the evaluation of marketing stimuli.

In the following paragraphs we intend to give a survey about the neuromarketing research recently published that used EEG and MEG methodologies. 

In particular, we attempt to describe the correlations observed in published literature between the EEG and MEG signals and particular aspects of the perception of marketing stimuli, such as the memorization of part or the entire sequence of the stimuli, as well as the perceived pleasantness of such stimuli. We also describe how EEG techniques could be used to assess changes in attention related to cultural differences in Western (Italy) and Eastern (China) population during the fruition of a commercial advertising.

## 2. Magnetoencephalographic TemporalPatterns of Cortical Activity

### 2.1. Product Choice

In this context, the research team of Sven Braeutigam [11–13] has employed the MEG in order to study the temporal relationship of cerebral areas involved in consumers' choices when they have to make decisions among different items within a laboratory context. 

In this study they wanted to analyse the cerebral behaviour by distinguishing male subjects from females during a simulated shopping. 

Cerebral activations induced by multiple choices reflect the level of familiarity or the preference that a particular experimental subject had with the presented products. These factors can be considered by taking into account the relationship between the current choice of a product on the shelf and the relative frequency of choice and usage of that product in the past. 

**Figure 1:**
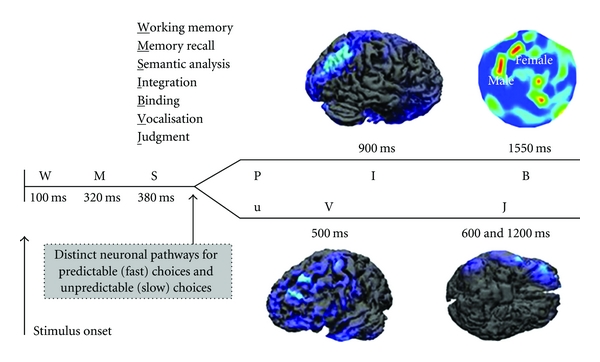
Cortical activations associated to the decisions of an experimental subject. Predictable choices (P) are the ones related to familiar items which have been often bought or used in the past, compared with the unpredictable ones (U). Label letters indicate the different stages of the trial, namely, working memory, memory recall, semantic analysis, integration, binding, vocalization, judgement, as the legend indicates as well. Cortical maps present the brain areas activated (blue: lower activity; red: higher activity) during the different decision stages in the frequency range from 30 to 40 Hz. Activations over the left prefrontal cortices are genderrelated, as the bidimensional map shows (high perspective, nose up), reproduced with permission from [11].

In particular, the main observation came to light from these studies presents the consumer's choice like a complex sequence of cerebral activations that greatly differ according to the consumer's sex and to the probability of choice. From a behavioural point of view, choices with a high probability were faster than those less predictable. This can be interpreted by supposing that in the case of more difficult choices the cortical activities are more complex than those simple to make. As illustrated in Figure 1, they distinguished two distinct cerebral paths. The first one is referred to predictable choices, that is, associated to products that the experimental subject already used in the past or said to prefer; the second one is related to unpredictable choices, that is, associated to unfamiliar products to the subject. In the experiment performed by Braeutigam, the first stage in the decisional process has been individuated around 100 ms after the stimulus onset with an activity located in the occipital cortex. At that stage of decision (working memory: W) the subject compared the product to choose with the list of products seen before by involving the working memory, although this early component is also usually related to the processing of sensory percepts. 

The sequence of cortical activations observed in the experimental subjects continues with two neuronal stages partially correlated (memory recall: M; semantic analysis: S) which can be observed between 280 and 400 ms after the beginning of the decisional process. In this period the selective attention of the subject is oriented towards images of products to identify, classify, and compare with those stored in the memory related to the preferred products and brands. This memory can involve the past experience of buying the particular item or watching the commercial of the specific brand.

### 2.2. Gender Differences

The cerebral activation differs in this time interval between men and women. In particular, around 400 ms after the stimulus onset, female subjects presented a stronger activation with respect to the males in the left parieto-occipital lobe of the brain while males presented a stronger cortical activity in the right temporal lobe, although authors do not show such results. These differences connected with the sex of the subjects characterize both the stage of choice of the product and its discrimination. These observations suggest that, at this temporal stage, women tend to employ a strategy based on the knowledge of the product to buy, while men tend to act according to a spatial memory strategy [14]. In addition, we could also say that men and women act according different semantic interpretation of the stimuli observed.

After 500 ms from the beginning of the decisional process, two patterns of cortical activations can be identified according to the predictability of the choices adopted by the subjects. In particular, as to the predictable choices (P), we can observe a strong activation in the right parietal areas around 900 ms after the beginning of the experiment (integration: I). In later time latencies, predictable choices of products recall strong MEG oscillations in the frequency band between 30–40 Hz in the left prefrontal cortex (binding: B). Parietal cortex receive inputs from many cortical areas since it is involved in the spatial integration of sensorial information. Differences in the cortical activity between men and women can strengthen the hypothesis of two different groups of strategies. On the contrary, unpredictable choices (u) generate a strong activation in the right inferior frontal cortex (vocalisation: V), at a latency of around 500 ms, and in the left orbitofrontal cortex (judgement: J) between 600 and 1200 ms after the stimulus presentation. In the case V, the cortical patterns are consistent with the activity in the Broca's area, which is involved in the spoken language, also active during the observation of video clips. Hence, the cortical activity at this latency may indicate a tendency to vocalize brands, as a part of strategy which helps in the decision when it is difficult. The activity in the orbitofrontal cortex (J) can be explained by stating that during an unpredictable choice we have to evaluate the outcome in terms of convenience. Overall, these results present a complex neuronal network which is active during a simple decisional process connected to the purchase of a product. The generation of a choice is considered as an information processing which can be highly influenced, sensible to the complexity of the decision to make and to the rush in which the decision is made along with many other factors.

### 2.3. TV Advertising

A strong involvement of parietal areas during the observation of the TV commercials with an affective and cognitive content was also noted in a previous study, performed by using sophisticated MEG recordings [15]. In that study, cognitive frames elicited a stronger activity in the parietal areas and superior prefrontal cortex while the observation of the affective ones is correlated with the activation of the orbitofrontal and retrosplenial cortex, amygdale, and brainstem. The magneto field tomography (MFT) results showed an increasing activity during the observation of cognitive stimuli rather than affective commercials in parietal and superior prefrontal areas. These regions are known to be associated with executive control of working memory and maintenance of highly processed representation of complex stimuli [16]. Although the affect-related activations are more variable across subjects, these findings are consistent with previous PET and fMRI studies [17–20] showing that stimuli with affective content modulates activity in the orbitofrontal and retrosplenial cortex, amygdale, and brainstem.

## 3. Electroencephalographic Frequency Patterns Cortical Activity and Functional of Connectivity

### 3.1. TV Advertising

**Figure 2:**
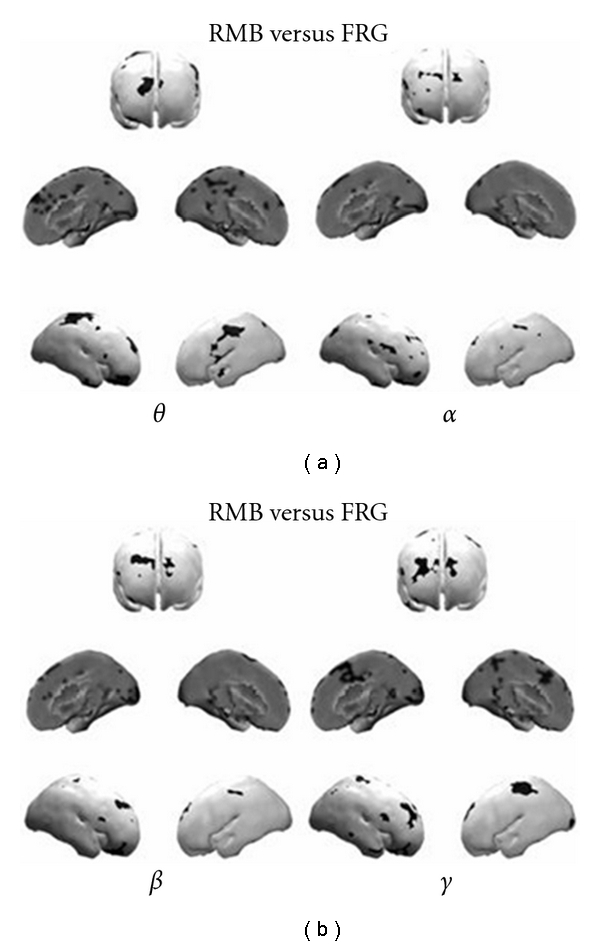
Figure presents the results of statistical comparisons of RMB and FRG groups in the theta and alpha frequency ranges (a), and in the beta and gamma bands (b). In particular, the picture is composed by three rows: the first one shows the brain from a frontal perspective, the second one is related to a medial-sagittal perspective, while the third one presents statistically significant images associated to the left and right lateral vision. Statistically significant increases of PSD values for the RMB group are represented as black spots on the average cortical model, reproduced with permission from [25].

The research in the neuromarketing field performed in the recent years by using high-resolution EEG techniques [21–24] showed also result suggesting that the cortical activity elicited during the observation of the TV commercials that were forgotten (FRG) is different from the cortical activity observed in subjects that remembered the same TV commercials (RMB). In fact, the principal areas of statistical differences in the power spectral density (PSD) maps, between such experimental conditions, are located almost bilaterally in the prefrontal Brodmann Areas (BAs) 8, 9 as well as in the parietal BAs 7, as shown in Figure 2. 

The spectral amplitude in the RMB condition was always higher than the power spectra in the FRG conditions over the BAs 8, 9, and 7 [25]. A statistical increase of PSD in the prefrontal and parietal areas for the RMB dataset compared with the FRG one is in agreement with the suggested role of these regions during the transfer of sensory percepts from short-term memory to long-term memory storage. Although in that study the differences in the cortical power spectra between the RMB and FRG conditions are relatively insensitive to the particular frequency bands and hemispheres considered, there are experimental evidences showing that there is a stronger engagement of the left frontal areas among all the subjects analyzed during the observation of the TV commercials that were remembered [26]. In particular in [26], the analysis of the statistical cortical maps in the condition RMB versus FRG suggested that the left frontal hemisphere was highly active during the RMB condition, especially in the theta and gamma band. These results are in agreement with different observations on the RMB condition performed in the literature by studying different experimental paradigms [17, 27]. Taken together, the results indicated that the cortical activity in the theta band on the left frontal areas was increased during the memorization of commercials. These results are in agreement with the role that has been advocated for the left pre- and frontal regions during the transfer of sensory percepts from the short-term memory toward the long-term memory storage by the HERA model [28, 29]. In fact, in such a model the left hemisphere plays a key role during the encoding phase of information from the short-term memory to the long-term memory, whereas the right hemisphere plays a role in the retrieval of such information. It must be noted, however, that the role of the right cortices in storing images has been also recognized for many years in neuroscience [14–17]. It is worthy of note that the subjects were unaware about the kind of questions that the researcher asked them after the viewing of the documentary. Hence, the cortical areas elicited by this study are likely to be involved just in the process of the memorization of the pictorial material, owing to an increase of attention during the observation of the TV commercial. In addition, there was no particular set of commercials remembered that was common to all the subjects. The pattern of activity, which was elicited during the observation of the TV commercials remembered after ten days, also suggest an active involvement of the anterior cingulate cortex (ACC) and the cingulate motor area (CMA) acting as sources of links going towards other cortical areas. In this case the increased activity related to an enhance of the outflow of partial directed coherence (PDC) links from ACC and CMA towards other cortical regions could be taken as a sign of increased “emotive” attention to the stories proposed by different TV commercials that significantly aid successive memorization. 

The EEG spectral and cortical network analyses performed in these study also suggest a key role of the parietal areas as targets of the incoming information flow from all the other cortical areas. Functional networks in the frequency domain were also estimated by evaluating the global- and local-efficiency indexes derived from the graph theory, employed as a measure of the level of communication in the networks [30–32]. The changes of these indexes could be related both to memory coding activity as well as to increase/decrease of attentive state of the subjects. As to the RMB condition, the functional network in the beta and gamma band state a significant nonhomogeneous allocation of the involved information flows and a consequent reduction of the efficiency in the overall communication between the network nodes. In the beta and gamma frequency bands, the respective reduction of global efficiency, as well as the reduction of local efficiency for the alpha band of the cortical network communication could represent a predictive measure for the accurate recall of the commercials that will be remembered.

### 3.2. Steady-State Visually Evoked Potentials

These results are also supported by findings obtained from the group of Richard Silberstein which measured the steady-state visually evoked potential (SSVEP) by means of the steady-state probe topography (SSPT), which is a particular version of the EEG technology [33–35]. In a particular study [33], they collected the cerebral activity from thirty five women that were subjected to the exposition of eighteen-minute documentary in which 12 US TV commercials were inserted within. Seven days after the recording, the participants were asked to recall the viewed advertisements from a series of frames taken from the same commercials. They found out that images corresponding to a minima of the posterior frontal latency were more likely to be recognized than images associated to a SSVEP latency maxima. Moreover, they showed a significant correlation between the recognition performance and SSVEP latency measured at electrode sites located in the left posterior frontal site suggesting that this kind of result can be employed in order to assess the strength of long-term memory encoding for the audiovisual stimuli they proposed. In this paper the main idea is then to use the particular cerebral probe adopted (as ms delays in the SSSVEPs) to assess the capability of a population to retain in the long-term memory part of the already observed advertisings. The practical value of a such kind of probe in marketing research is linked to an automatic evaluation of the “memorability” of advertising, or even part of it. The interest of marketers for this information is related to the fact that it is quite sure that an advertising that cannot be remembered does not help so much in the selling of the product, while not necessarily it is true the vice versa.

### 3.3. Hedonic Evaluation of Logos

The research group of Handy and colleagues, instead, shifted their attention towards the rapid and emotional evaluation of advertising logos [36]. Their study want to inspect whether the visuocortical processing of everyday images, like logos, can include an implicit hedonic analysis. In particular, they asked participants to identify, within a set of unfamiliar logos, those that were most liked or disliked. By means of an event-related potentials (ERPs) analysis, they found out that visuocortical processing shows an increase of the early positive component (named P1), at central and parietal sites, along with an increase of the later negative component (named N2), at parietal and occipital sites, related to the observation of disliked logos. The idea at the base of this paper is to find electrophysiological signs correlated to the perception of liking or disliking particular advertising logos. Once this correlation was found, as expressed in the paper, it opens the way to the use of such ERPs P1 and N2 waves as markers for the hedonic preferences of the consumer concerning the logos. This procedure could overcome the need to collect verbal preferences of consumers during the evaluation of different kind of logos, by replacing them with an automatic and nonverbal evaluation of such hedonic preferences. Probably these information, related to N1 and P2, could be sufficient in the analysis of a simple logo while other type of variables, linked to more complex experiences by the users, could be employed to assess pleasantness of more complex marketing stimuli, such as an entire product (with the logo included). However, the possibility to have nonbiased clues about the “inner” perception of simple symbols in the brain of the consumers could have a marketing value and it will add a piece in the puzzle of the comprehension of the complex relationships between brand and consumers.

### 3.4. Pleasantness and Frontal Asymmetry

The recognition in humans of positive or negative hedonic values during theobservation of different logos or advertising could be assessed also byusing EEG spectral activity instead the ERPs. In fact, with the use of EEG rhythms indirect variables of emotional processing could be gathered by tracking variations of the activity of specific anatomical structures linked to the emotional processing activity in humans, such as the pre- and frontal cortex (PFC and FC, resp.; [37]). It is known that the PFC region is structurally and functionally heterogeneous but its role in the generation of the emotions is well recognized [38]. EEG spectral power analyses indicate that the anterior cerebral hemispheres are differentially lateralized for approach and withdrawal motivational tendencies and emotions. Specifically, findings suggest that the left PFC is an important brain area in a widespread circuit that mediates appetitive approach, while the right PFC appears to form a major component of a neural circuit that instantiates defensive withdrawal [39, 40].

A contrast of the activity elicited by observing pleasant (LIKE dataset) and unpleasant (DISLIKE dataset) audiovisual content has been performed in a previous study [26]. The result of this experiment shows that the activity of the brain, in terms of PSD, is stronger in the LIKE condition than in the DISLIKE one except that in beta band. The results here obtained for the LIKE condition are also congruent with other observations performed with EEG in a group of 20 subjects during the observation of pictures from the international affective picture system (IAPS, [41]). Such observations indicated an increase of the EEG activity in the theta band for the anterior areas of the left hemisphere.

**Figure 3:**
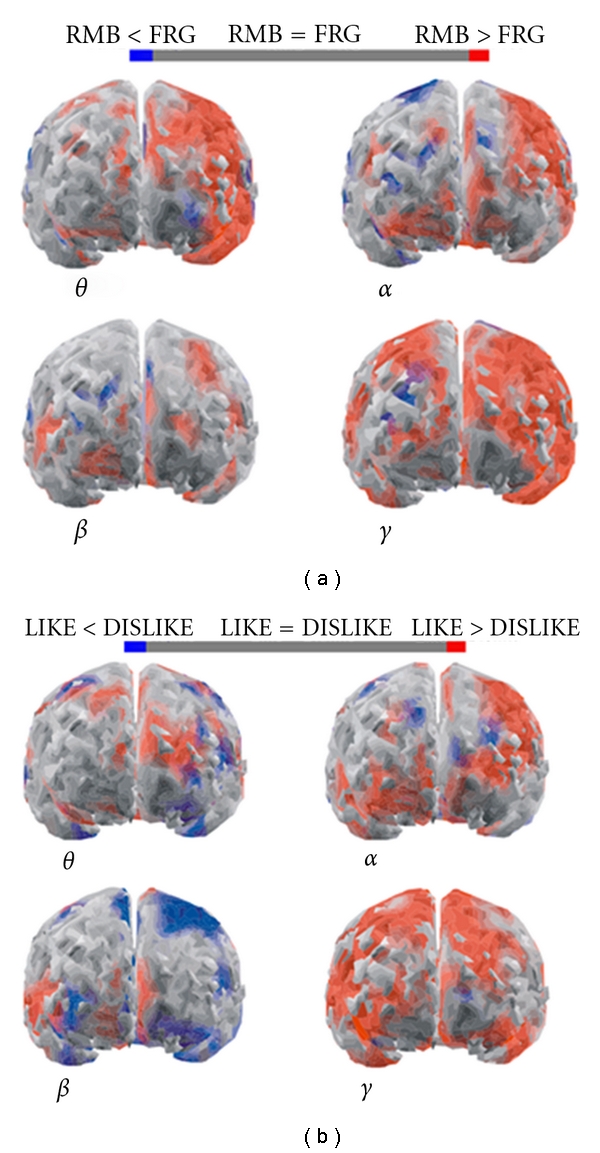
Figure presents four cortical *z*-score maps, in the four frequency bands employed. The average cortical model is seen from a frontal perspective. Colour bar represents cortical areas in which increased statistically significant activity occurs in the RMB group when compared to the FRG group in red, while blue is used otherwise (*P* < 0.05 Bonferroni corrected). Grey colour is used to map cortical areas where there are no significant differences between the cortical activity in the RMB and FRG groups (a). (b) refers to the statistical comparison LIKE versus DISLIKE with the same conventions of (a), reproduced with permission from [26].

Figure 3 also highlights the activation of the ACC which is an area involved in reward-based learning and error detection. According to this knowledge, we may also suggest that reward-based learning could be playing a part when observing pleasant and unpleasant advertisements. 

**Figure 4:**
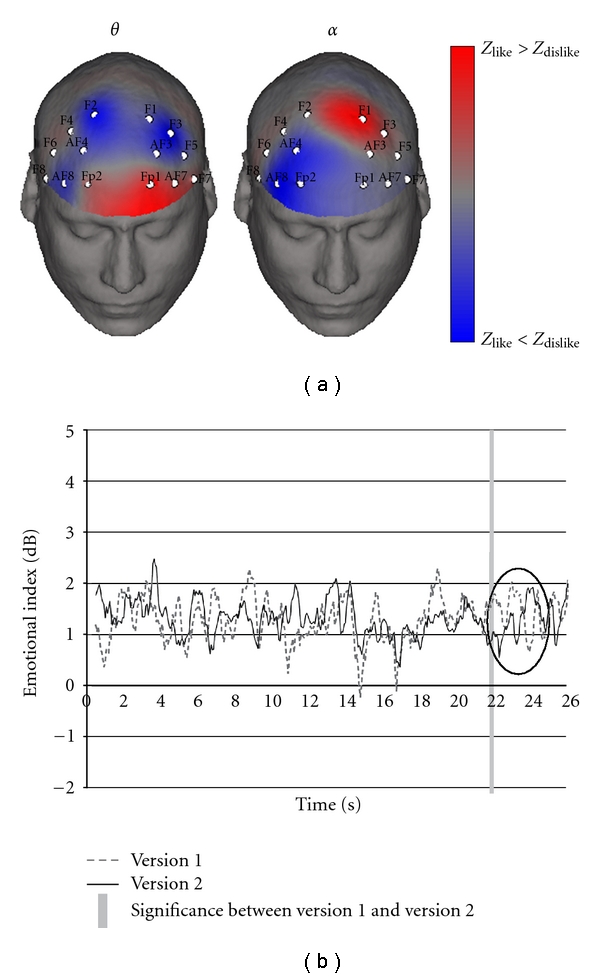
(a) The two scalp maps in figure represent the *Z* score distribution for the theta (left) and alpha band (right) in the population analyzed. *Z* values are mapped onto a realistic scalp model, seen from a frontal perspective. Colorbar codes scalp areas in which the LIKE spectral activity is greater than the DISLIKE (red) and regions in which the DISLIKE spectral activity is greater than the LIKE (blue). Grey indicates regions with no difference between the two experimental conditions, reproduced with permission from [42]. (b) Electroencephalography trace of emotional response. In version 1, only the model's face was presented; conversely, in version 2, the viewers saw her face from a slightly different angle (for 2.5 s), and then she made a particular manual gesture (for 1.5 s), reproduced with permission from [43].

In order to deeper investigate such a phenomenon, in a recent work we calculated a spectral imbalance at frontal electrodes for EEG dataset related to spots the subjects judged pleasant (LIKE dataset) or unpleasant (DISLIKE dataset). We observed that such LIKE and DISLIKE datasets are characterized by different EEG power spectral maps in theta and alpha bands [42]. In particular, the activity in the left frontal hemisphere is related to the observation of commercials that have been judged pleasant by the analyzed population (Figure 4(a)). On the other hand, the right frontal sites highlighted neuroelectrical activations concerning the observation of advertisements that have been judged less pleasant by the same population. Moreover, this imbalance in the activations was linearly correlated with the degree of pleasantness expressed by the subjects. Overall, the right frontal activity is significantly greater than the one in the left frontal lobe, both in theta and alpha bands. All together, these results are in line with previous findings suggesting the presence of an asymmetrical EEG activity when subjects experienced emotional stimuli [37, 38].

By employing the same theoretical background, Polish researchers performed a particular advertisement before test concerning the analysis of a skin care product [43]. They found out that the observation of two versions of the same TV commercial, differing only for a very short scene containing a particular gesture by a female model (from 21st to 25ths), generated significantly different emotional impact. In accordance with the Davidson's model, they measured the emotional response of the advertisement by calculating the difference in the amount of the alpha activity between the left and the right hemisphere taken from frontal electrodes, defined by them as emotional index. By analysing the difference of emotional index, for each second of the TV spot, elicited from the observation of the two ad versions, they found out a significant difference during the 22nd second. In particular, the scene showing only the model's face evoked a more positive emotion than the scene presenting both the model's face and her hand gesture, while no significant difference was found in other segments (Figure 4(b)). 

Taken together, these mentioned examples bring evidences that it is possible to link some properties of the collected EEG rhythms during the watching of some TV advertisings with the overt preferences of the observers in terms of emotions. This link, as in the example we provided with the event-related potentials (ERPs), could be used to generate a metric that automatically points to parts of the advertisings examined that are emotionally OK and parts that are not. These information could be used “*a posteriori*” to redraw partially the advertising in order to increase the appearance of the “like” parts while depressing the “dislike” parts. Such reduction or modification of the broadcasted TV advertisings could be then performed by using EEG techniques thanks the high time resolution provided by this methodology.

## 4. Tracking Cultural Differences in Advertising between Western and Eastern Users: An Example of the Neuroimaging in Advertising

**Figure 5:**
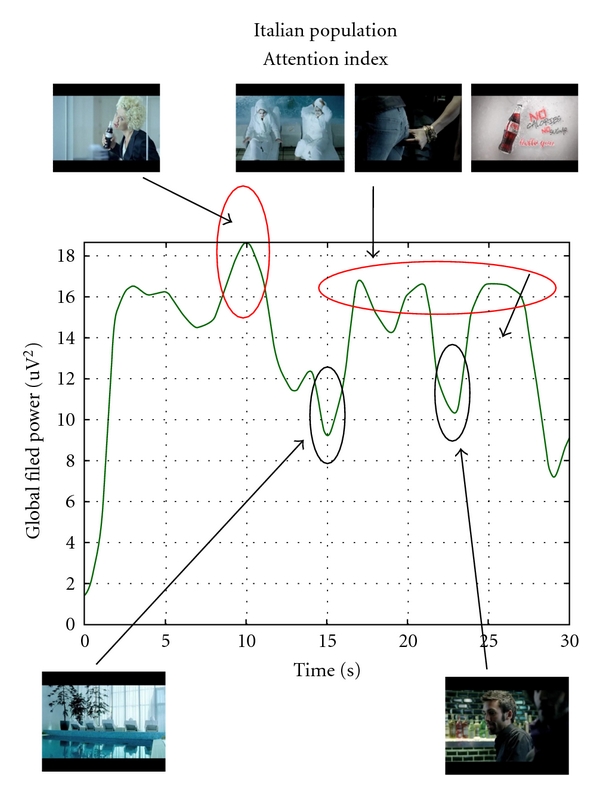
Representation of the filtered and smoothed GFP related to the frontal electrodes in the lower alpha band for the analyzed Italian subjects during the observation of the TV commercial advertising a carbonated beverage. On the *x*-axis, there is the time duration of the spot; on the *y*-axis there is the *z*-score smoothed values of the GFP considered. Film segments are showed when increase of cerebral activity occurred.

**Figure 6:**
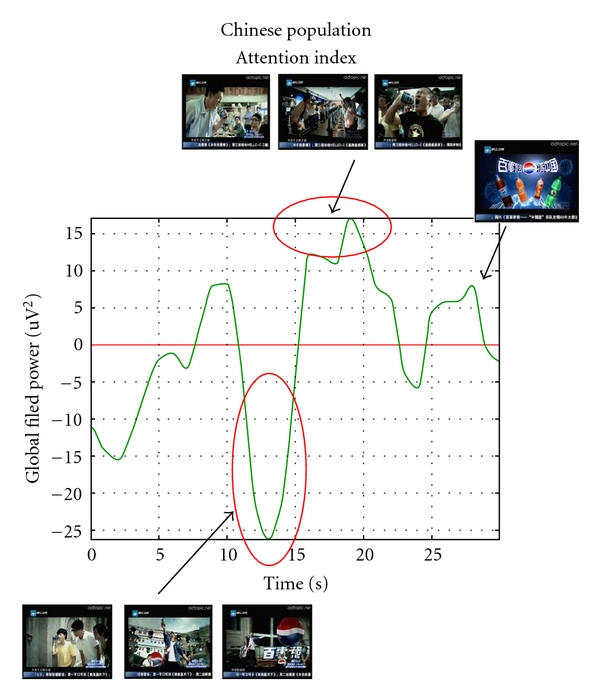
Representation of the filtered and smoothed GFP related to the frontal electrodes in the lower alpha band for the analyzed Chinese subjects during the observation of the TV commercial advertising a carbonated beverage. On the *x*-axis, there is the time duration of the spot; on the *y*-axis there is the *z*-score smoothed values of the GFP considered. Film segments are showed when increase of cerebral activity occurred.

Advertisings related to the fruition of carbonated beverages are intensively presented on the usual TV programs worldwide. Such carbonated beverages advertisings are also present on journals, street posters, fast foods, and supermarkets in a pervasive way and could have a deep impact on human behaviour. Recent functional neuroimaging studies have reported activity in ventral and/or medial prefrontal cortex during the contemplation or consumption of familiar brand-name products [2, 44, 45]. Since lesion studies indicate ventromedial prefrontal cortex (VMPC) is critically involved in emotion, emotional regulation, and decision-making [24, 46, 47], ventromedial prefrontal activations can be interpreted as evidence for emotion playing a pivotal role in brand preference [44, 45]. While the role of prefrontal cortices is then highlighted in the generation of appreciation for a brand, it is not really addressed the issue how this appreciation is spread across different cultural models, that is, across different Western and Eastern people. In fact, it is well known that the different cultural model in Western and Eastern culture leads to different appraisal of the same experience or situation. Hence, it is of value to understand if people educated in different culture could react to the same kind of advertising related to carbonated drinks in a similar way from a cerebral point of view. Here we briefly reported a study aimed to track cerebral activity during the fruition of similar advertising related to very popular carbonated beverages (Coca Cola and Pepsi Cola) in group of Western and Eastern people, homogeneous for age. In particular, we conducted two series of experiments in Italy and China aimed to the collection of EEG data related to the fruition of Coca Cola and Pepsi Cola advertisings. Analysis was then focused on the activity on the prefrontal cortices during the fruition of such video clips in both populations. EEG recordings were performed on a population of 15 healthy volunteers in Italy and 13 healthy volunteers in China. The procedure of the experimental task consisted in observing a twenty-minute long documentary in which we inserted different advertising breaks. Each interruption was formed by the same number of commercial video clips of about thirty seconds. In such a case, during the whole documentary, a total of twenty-four TV commercials were presented. Advertisings used are related to standard international brands of commercial products, like cars, food, and so forth, but for the following comparison we will only show the results belonging to the exposition of a TV commercial advertising presenting a popular international brand of two different carbonated beverages. The neurophysiologic activity gathered during the observation of the documentary was also analyzed as a baseline (REST dataset). Although we are aware that the personal involvement, and the related cerebral activity, also depend on the topic of the documentary, we believe that this realizes a lifelike situation where people watche advertisements usually within a movie. Consequently, their level of attention will depend both on the commercial content and on the context which it is inserted into. Each EEG trace has been bandpass-filtered in order to isolate the only spectral components in the theta and in the lower alpha bands from the whole EEG spectrum. These filtered theta and lower alpha traces have been employed to calculate the Global Field Power (GFP; [48]) for each segment, then converted in *z* scores in order to extract cerebral indexes following described. Since for the phenomena we would like to investigate a clear role of the frontal areas that have been depicted [16, 27] we used the frontal electrodes to compute the GFP indexes used in the following. In order to synthesize the properties of the cerebral activation for the analyzed ads we used the lower alpha bands to define an Attentional Index [49]. The filtered EEG signals were subjected to the computation of the Global Field Power by taking into account the signals that comes from the frontal and prefrontal electrodes of the 10-10 International System: F3, F4, AF3, AF4, F7, AF7, F8, AF8, Fz, AFz. In the following figures, time-varying changes of the filtered and smoothed GFP in the lower alpha bands during the observation of both Italian (Figure 5) and Chinese (Figure 6) TV commercial advertising of a carbonated beverage are shown. In Figure 5 it is possible to observe that the attentional level of the Italian subjects, during the observation of the commercial, is above the zero level elicited during the REST period for the whole length of the videoclip. The videoclip is related to a series of short stories in which the presence of Coca Cola makes the actors happy. The spot closes with the presentation of the brand and with the product. The level of attention during the observation of the Italian spot is generally high for the whole length of the spot. In particular, there are film segments of high attention around the 10′′ when a girl, holding the advertised beverage, is making jokes with her face and when guys are having fun in a party (showed around the 20′′).

Slight decreases of attention occur when a pool is showed (15′′) and when the scene turns on guys in a pub (23′′). The level of attention raises again during the final seconds of the commercial. 

In Figure 6 we show the results obtained by analyzing the cerebral activity collected among the Chinese dataset. The video presented is related to the young male that uses the Pepsi can as a microphone. Successively, when he sings different persons around a city could listen to him through their own Pepsi can. There is an alternation of scenes in which the young male sing alone and groups of persons listen to and sing by using the same Pepsi can. The spot closes with the presentation of a series of Pepsi products.

The attentional index presents three peaks of frontal cerebral activity roughly located around the 10′′, 20′′, and 28′′ of the advertisement. Such parts are related to the frames in which the echoes of the music generated by the singer is very low (10′′), and when multiple people start to sing together, respectively (20′′, 28′′). Hence, it seems that the frames in which activity from multiple people is represented get the maximum of attention from the Chinese group investigated. On the contrary, it is possible to distinguish at least three negative peaks around the 2′′, 14′′, and 24′′ of the same commercial. Such frames are all presenting the singer alone, who sings on a stage. The last increase in attention occurs when the logo, brand, and products from Pepsi Cola appears on the screen. 

Summarizing the results of the presented experiment, differences in the time course of the average attention generated in the groups related to the fruition of the video have been observed. Such differences in both populations are likely generated by the different plot of the commercial advertisings presented. However, some invariant cerebral activity in frontal cortices could be noted related to the presentation of the brand (Coca Cola or Pepsi Cola) of the carbonated beverage. In fact, both groups react in the same way to the presentation of the brand (different for the Italian and for the Chinese population), that is, with a peak of attention located in different moments of the commercials. In the case of the fruition of Pepsi Cola in the Chinese group, the greatest peaks of attentions are obtained in visual scenes involving a large number of persons, while the lowest scores of attention are obtained by frames in which the singer was on the stage to play the guitar. In the case of fruition of Coca Cola advertisement in the Italian analyzed group, the major impact of the advertising was when a single subject performs jokes to the other and when the brand was presented. Social cognition research differentiates cultural contexts that emphasize ideas and practices of interdependence (e.g., East Asian cultures in China, Japan, and Korea) from those that emphasize ideas and practices of independence (e.g., Western contexts in North America and Western Europe; [50, 51]). These cultural differences were originally considered in terms of social relations, but subsequent research has shown that they also apply to performance of simple perceptual judgments. Cultural differences in the frequencies of certain types of expressions and behaviors tend to reflect differences in cultural models as well. East Asian cultural models, for example, stress relational harmony and promote that individuals take their proper place. These cultural models discourage individuals from occupying too much space in the relationship, both figuratively and literally. Thus, expansive behavior, such as general somatic activity, is a signal that the individual is taking more than his/her proper space. 

The results of this study seems to support the observation that both groups (Chinese and Italian) tend to be very sensitive to the presentation of the brand of the beverage, while Chinese group is not sensitive to the presentation of frames in which a unique actor is dominating the stage, while this is the contrary for the Italian group. These results suggest that while the brand of carbonated beverages attracts the attention of both groups, cultural differences between such groups were reflected in a different span of attention when the performance of a unique actor or an ensemble of them was observed. Chinese group follows with more attention the collective scenes, while Italian group follows with more attention scenes in which a unique actor is performing, although we should specify that we did not control the luminance level of the two advertisements, which could also play a role in the increase of attention. In future experiments, it becomes of interest to analyze the two populations when experiencing the same commercial stimuli.

## 5. Conclusions

In recent years, neuromarketing has gained always more interest and attention in both the scientific community and mass media.

However, it is important for the scientific and marketing communities to get an answer to a series of questions related to the use of neuroscience in marketing research. In particular, it is important to understand which could be the practical use of such kind of neuroscience research in marketing, and how it can be bettered in future research. In fact, at the moment the neuroscience have returned a series of correlations between different properties of the neurophysiologic cerebral signals and the nature of the advertising stimuli. 

From the point of view of memorization processes, it has been suggested as delayed responses of the SSVEP could be a probe for the memorization on long-term memory performed during the observation of a commercial advertising. Or as the topology of an estimated cortical network by EEG could be strictly related to the memorization on a medium-term memory still of a commercial videoclip. If these signs of memorization are useful in marketing research it is an interesting question. The added value of this information, when compared to the verbalization of the subjects after their exposition to the commercial videoclip is twofold: (1) the subjects could not state the truth verbally or could be even unaware of particulars that they could remember from the clip; (2) since the memory process could be tracked with the EEG (MEG) technique on a msec base, hence a clue on which particular scene of the videoclip is working and which is not, could be easily obtained. This is clearly an added value when compared to the poor time resolution provided by the fMRI applied in this neuromarketing context. It could be anticipated here that the capability to track memorization processes of a TV spot with a time resolution of 1 second makes possible to detect if such processes are statistically significant during the exposition of the brand or simply during the entire spot per se. In this last case it will mean that the spot will be remembered but not probably the brand, while in the first case we will remember just the brand but not the spot.

From the point of view of the track of emotional processes, reviewed papers have suggested that particular signs of brain signals, such as deflections called N1 or P2 in the ERPs, or asymmetries in spectral power in alpha and theta bands on frontal areas, could be related to the emergence of pleasantness or unpleasantness regarding the perceived marketing stimuli.

Overall, results presented in this paper also highlight that the left prefrontal cortex is activated both while subjects are observing stimuli that will be later remembered and by the ones which will be judged pleasant. In this way, the prefrontal cortex plays a key role in the neuromarketing research since the neural activity in these areas seems to discriminate both a load of cognitive processes, such as the encoding of new complex stimuli (e.g., logos, products, testimonials, payoff, etc.), and variations of the emotional state of the subject.

Actually, such electrophysiological signs were again used as a probe to assess pleasantness of such commercial stimuli, supporting or even replacing the verbalization of the subjects after the observation of the commercials. Again, if the perceived pleasantness of the commercial videoclip could be a critical quality for the assessment of a brand or for the promotion of a product, it is an issue that goes beyond the aim of this discussion.

As we showed above with several examples, we believe that the nowadays technology is ready to provide useful information to marketers by employing neuroimaging tools. In fact, we illustrated how, thanks to the brain imaging, we are able to track the cerebral activity and investigate which parts of an advertisement can elicit an emotional reaction and which ones cognitive processing. Since these kind of techniques allow looking to see if people remember/forget an advert, what attention they are giving, whether they like/dislike it, and whether they evaluate it cognitively/emotionally, marketers could exploit these tools in an ad pretest in order to verify if the TV commercials they realized, or also particular key frames, elicit the emotion or the cognitive engagement they established in advance.

A drawback of the experiments presented lies in the fact that, among researchers working in neuromarketing, there is not a common and shared experimental paradigm yet. This field of research is newborn and it seems to need some more time to strengthen its basis. Although they are well recognized in the international scientific community, neuromarketing researchers still need to share their knowledge and findings. This would lead to better define experimental paradigms which will later provide a common light to interpret results and to pave the way to future research. 

However, the application of the today neuroscience techniques to marketing stimuli can be of help for many areas of marketing. For instance, information gathered in this way could be employed both during the design process of a product and during its commercial campaign to advertise it. In the first case, the information gained by using brain imaging could be used to further tune the characteristic of the product. In the latter case, such information will be used to understand the adequacy of the proposed advertising campaign to the message the particular industry would like to convey to the public. The result of this entire process will be a product that could match better the demand of the consumers.
